# Can Schlafen 11 Help to Stratify Ovarian Cancer Patients Treated with DNA-Damaging Agents?

**DOI:** 10.3390/cancers14102353

**Published:** 2022-05-10

**Authors:** Marketa Bednarikova, Jitka Hausnerova, Lucie Ehrlichova, Kvetoslava Matulova, Eliska Gazarkova, Lubos Minar, Vit Weinberger

**Affiliations:** 1 Department of Internal Medicine, Hematology and Oncology, Masaryk University and University Hospital, 625 00 Brno, Czech Republic; bednarikova.marketa@fnbrno.cz (M.B.); ehrlichova.lucie@fnbrno.cz (L.E.); 2 Department of Pathology, Masaryk University and University Hospital, 625 00 Brno, Czech Republic; hausnerova.jitka@fnbrno.cz (J.H.); matulova.kvetoslava@fnbrno.cz (K.M.); 3 Department of Obstetrics and Gynecology, Masaryk University and University Hospital, 625 00 Brno, Czech Republic; gazarkova.eliska@fnbrno.cz (E.G.); minar.lubos@fnbrno.cz (L.M.)

**Keywords:** SLFN11, ovarian cancer, high-grade serous carcinoma, DNA-damaging agents, PARPi, chemoresistance

## Abstract

**Simple Summary:**

Anticancer agents causing DNA damage are widely used in the treatment of ovarian cancer; however, predictive biomarkers capable of optimizing the selection of patients with the best likelihood of a good response have not been defined yet. The newly discovered protein Schlafen 11 was identified to have a casual association with responses to a wide range of DNA-damaging agents, including platinum compounds and PARP inhibitors. We review the current state of knowledge regarding the role of Schlafen 11 as a biomarker in ovarian cancer and its potential to identify patients not responding to the systemic treatment.

**Abstract:**

Platinum-based chemotherapy has been the cornerstone of systemic treatment in ovarian cancer. Since no validated molecular predictive markers have been identified yet, the response to platinum-based chemotherapy has been evaluated clinically, based on platinum-free interval. The new promising marker Schlafen 11 seems to correlate with sensitivity or resistance to DNA-damaging agents, including platinum compounds or PARP inhibitors in various types of cancer. We provide background information about the function of Schlafen 11, its evaluation in tumor tissue, and its prevalence in ovarian cancer. We discuss the current evidence of the correlation of Schlafen 11 expression in ovarian cancer with treatment outcomes and the potential use of Schlafen 11 as the key predictive and prognostic marker that could help to better stratify ovarian cancer patients treated with platinum-based chemotherapy or PARP inhibitors. We also provide perspectives on future directions in the research on this promising marker.

## 1. Introduction

Ovarian cancer (OC) is the leading cause of death amongst all gynecological cancers, with an overall 5-year survival rate of no more than 35% for advanced stages [1,2]. For decades, primary therapy has consisted of cytoreductive surgery and platinum-based chemotherapy (CT). The leading cause of the high mortality rate, besides the diagnosis at an advanced stage, is the sooner or later acquired resistance to systemic therapy despite a good response in the majority of cases initially. Thus, platinum sensitivity or resistance, together with tumor stage, histotype, and the ability of complete surgical resection, represent the main clinically used prognostic factors. Moreover, platinum sensitivity is the key predictive factor for the successful subsequent treatment of recurrent disease [3,4].

Lately, the anti-angiogenic agent bevacizumab, and poly(ADP-ribose) polymerase inhibitors (PARPi) have become an integral part of the systemic treatment of primary or relapsed ovarian cancer [5,6,7,8]. Unfortunately, the precise selection of patients with a high probability of a good response to this novel therapy remains far from optimal. Despite the high cost and significant toxicity of bevacizumab, no predictive markers for treatment response have been identified yet. The precise prediction of platinum resistance would help to protect non-responders from an even more aggressive combination with bevacizumab. Similarly, regardless of the known effect of PARPi, especially in ovarian cancer with either a mutation in the BRCA 1/2 genes or a deficiency in the homologous recombination system (HR), even patients with HR-proficient tumors may benefit from PARPi [9]. On top of that, not all BRCA-deficient tumors are sensitive to PARPi [10]. The sensitivity to PARPi has been associated with defects in the DNA damage response. Therefore, the identification of markers predicting sensitivity to platinum compounds belonging to DNA damage-inducing agents (DDA) could also help in this field [11,12].

So far, platinum sensitivity or resistance has been evaluated clinically, based on a platinum-free interval (TFIp), i.e., the time between the last course of platinum-based chemotherapy and the progression of the disease. Despite platinum-sensitivity in the majority of ovarian cancers, there is a significant proportion of patients with primary platinum-refractory or resistant disease (i.e., progressive during or shortly after the termination of primary chemotherapy). In other words, about 30–40% of patients undergo an ineffective initial treatment accompanied by substantial side effects [4,13,14]. Although some genetic alterations are known to be associated with resistance to the platinum-containing regimen, no validated molecular predictive biomarkers capable of predicting response to platinum-based chemotherapy have been identified yet [14,15,16,17].

Schlafen 11 (SLFN11), a member of the mammalian Schlafen family of growth regulatory genes first described in 1998, was recently identified to have a casual association with response to a wide range of DDA, including platinum salts and PARPi. Multiple preclinical models and some clinical studies have demonstrated that high SLFN11 expression levels positively correlate with increased DDA sensitivity in various types of cancers [11,18,19,20,21,22,23]. Conversely, the loss of SLFN11 expression is associated with resistance to these therapeutics [10,24,25]. Independent groups proved that SLFN11 expression in tumor cells could be easily assessed by immunohistochemistry [26,27,28,29]. Altogether, data published so far suggest the promising role of SLFN11 as a predictive biomarker in the clinical setting across multiple cancers [12,30].

In this review, we aim to summarize the current knowledge of the role of SLFN11 in ovarian cancer. Here, we briefly describe the function of Schlafen 11 and its vital importance for the maintenance of genomic stability under replication stress. We review the available information about the prevalence of SLFN11 in ovarian cancer, the possibilities of its determination in tumor tissue, as well as the current level of evidence about the correlation between SLFN11 expression levels in ovarian cancer and response to DDA or PARPi. We also focus on the available data on the potential prognostic role of SLFN11 in ovarian cancer patients.

## 2. Materials and Methods

For this review, we used the results of studies and review articles on the subject published in English up to December 2021. They were identified through a search of the literature using PubMed, MEDLINE-Ovid, Scopus, and Cochrane Library with the keywords (‘Schlafen 11’ OR ‘SLFN11’ OR ‘DNA damage’ OR ‘DDA’ OR ‘PARPi’ OR ‘platinum sensitivity’ OR ‘platinum resistance’) AND (‘ovarian cancer’ OR ‘high-grade serous carcinoma’). We retrieved and assessed potentially relevant papers and checked the reference list of all papers of interest to identify additional relevant publications.

## 3. SLFN11 as a Guardian of the Genome in Response to Replication Stress

In 2012, two independent research groups discovered by bioinformatic analyses of large cancer cell line panels that the nuclear protein SLFN11 is the causal and dominant genomic determinant of response to DNA-damaging agents [10,24]. Further studies confirmed that the nuclear protein SLFN11 plays a crucial role in cell cycle arrest and the induction of apoptosis in response to replication stress and therefore acts as a guardian of the genome. In case of various types of DNA damage caused by anticancer agents, such as covalent DNA adducts formed by platinum compounds or inhibition of DNA repair by PARPi (see Table 1), SLFN11 binds to stressed replication forks and thus blocks replicative helicase complex, induces chromatin opening, and forces the degradation of the key replication factor CD1 [31,32,33,34]. These processes ultimately lead to irreversible cell death.

SLFN11-mediated cell cycle inhibition works independently on the classical signal pathway of ataxia telangiectasia mutated and Rad3-related and checkpoint kinase 1 (ATR/CHK1) [36,37]. Notably, the effectivity of non-DNA-damaging anticancer drugs acting by a different mechanism (such as microtubules inhibitors, kinase inhibitors, or mTOR inhibitors) is independent of the level of SLFN11 expression [10,24].

**Figure 1:**
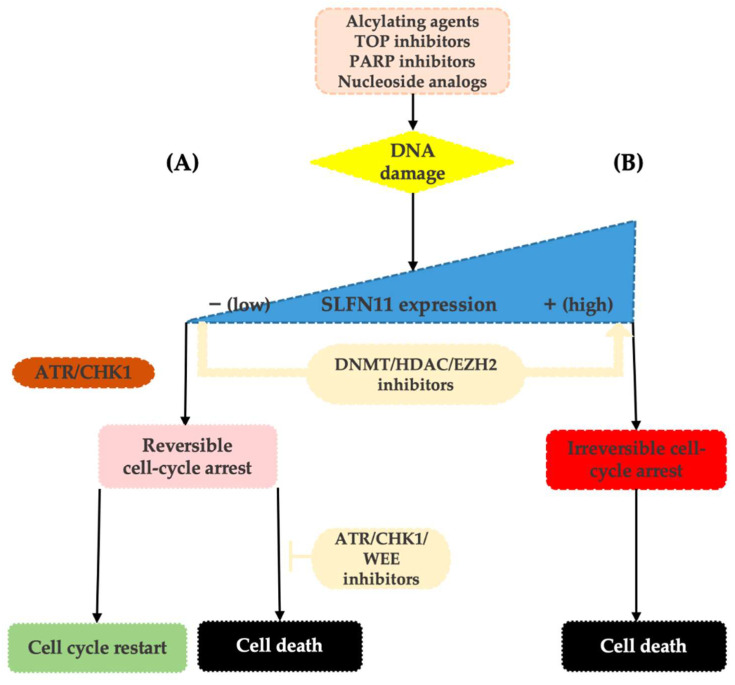
Upon replication stress caused by various types of DDA, SLFN11-proficient cells undergo an enforced G1/S arrest ultimately resulting in cell death (**B**). On the contrary, SLFN11-deficient cells reliant on the ATR/CHK1 pathway re-enter the cell cycle, slowly progress through the S-phase, and following DNA repair can survive (**A**). SLFN11 expression can be reactivated by the inhibitors of epigenetic modulators, such as DNA methyltransferase (DNMT), histone deacetylase (HDAC), or EZH2 inhibitors. Resistance of SLFN11-deficient cells can be overcome by combination with ATR/CHK1/WEE1 inhibitors.

Lack of SLFN11 expression makes the functionality of the SLFN11-mediated system impossible and increases tumor viability. SLFN11-low cancer cells then rely on the ATR/CHK1 DNA repair system that enables only reversible cell-cycle arrest in response to replication stress. Cells are then capable of repairing DNA damage and recover replication, which contributes to resistance to DDA [12,25] (see Figure 1).

Based on detailed analyses of cancer cell lines, neither clinically meaningful SLFN11 mutations nor its copy number variations have been reported. Thus, the current evidence suggests that the expression level of SLFN11 is largely regulated by epigenetic processes such as DNA methylation and histone modifications [36,38,39]. Indeed, in different tumor types, epigenetic modifications have been shown to cause loss of SLFN11 expression and associated resistance to DDA [25,36,39,40,41,42]. These epigenetic processes may also induce dynamic changes in tumor expression levels of SLFN11 during chemotherapy and cause the development of acquired resistance to DDA [23,43,44]. On top of that, as these modifications are reversible, the therapeutic interventions using inhibitors of DNA methylation (such as 5-azacytadine) or histone deacetylases (such as romidepsin or entinostat) may result in the re-increase in SLFN11 tumor expression levels and thus re-sensitization to DDA, making SLFN11 an attractive therapeutic target [25,34,42,43,45,46]. The other way to synergistically overcome the resistance of low SLFN11 tumors to DNA-damaging agents is by using replication checkpoint inhibitors (ATR/CHK1/WEE1 inhibitors), such as berzosertib, ceralasertib, and elimustertib [35] (Figure 1).

## 4. Clinical Evaluation of SLFN11 Status

Initially, quantification of SLFN11 expression either in cancer cell lines or patient-derived xenografts (PDX) was performed by transcript analyses using Western blotting [10,11,18,24,43]. Subsequently, protein assessment in tumor tissue by immunohistochemistry (IHC) was proven as an acceptable method in various types of cancer [21,26,27,28].

According to the study by Lok et al. on PDX models, SLFN11 IHC scoring seemed to be a stronger predictor of PARPi efficacy than both SLFN11 gene expression and protein expression by Western blot [27]. Subsequently, Takashima et al. demonstrated a substantial discrepancy between SLFN11 expression in tumor samples assessed by IHC and RNA-seq performed in non-microdissected tissue samples from The Cancer Genome Atlas (TCGA) datasets. Levels measured by RNA-seq reflected not only SLFN11 positivity in the cancer cell, but also in the surrounding stromal or inflammatory cells. On the other hand, immunohistochemistry enabled distinctions between positivity in cancer and non-cancer cells. These results indicate a potential of IHC to be of key importance for the precise identification of SLFN11-negative tumors that can be non-responsive to the DNA-damaging agents [29]. Therefore immunohistochemistry, the well-established and widely accepted pathological method, seems to be optimal for the evaluation of SLFN11 expression in patient samples. Undoubtedly the feasibility of immunohistochemical testing for SLFN11 could facilitate the rapid implementation of SLFN11 assessments into clinical practice.

One of the important studies aiming to provide a practical resource for the utility of SLFN11 in clinical practice was published by Takashima et al. They performed a comprehensive analysis of SLFN11 expression in malignant and the adjacent non-tumor tissues across 16 human organs. They compared three commercially available antibodies and proved the mouse D-2 (#sc-515071, Santa Cruz) had the best specificity and sensitivity ratio. They also established a rigid step-by-step protocol and scoring system for evaluating SLFN11 expression by IHC. According to the average values of the ratio of positivity, SLFN11 immunohistochemical scores were considered 1+ (1–10%), 2+ (11–50%), or 3+ (51–100%). Their results demonstrated a broad diversity of SLFN11 expression among organs. They also showed SLFN11 expression to be highly dynamic and very different in non-tumor and tumor tissues. While in some organs, such as colon and prostate, tumor and non-tumor tissues were consistently negative, in other organs there was a tendency for SLFN11 positivity to be higher (breast, uterine corpus, ovary) or lower (lung, glioblastoma, papillary renal cell carcinoma) in tumors compared to non-tumor tissues [29].

As far as experiences with immunohistochemical testing for SLFN11 in ovarian cancer are concerned, in addition to Takashima et al., the results of four other studies have been published so far. Firstly, Velone presented at the SLFN11 monothematic workshop in 2017 the analysis of 75 cases of high-grade serous carcinoma (HGSC). As there was no commercial kit available for SLFN11 immunohistochemical testing at that time, they adopted two kits originally commercialized for Western Blot. According to their findings, IHC appeared clean and specific in samples of HGSC. For the evaluation of SLFN11 levels by IHC, they used the histological score (H-score; HS) with values 0–9 calculated based on an evaluation of the staining intensity score (IS) and the distribution score of stained cells (DS). Cases were then considered as “SLFN11 negative” (HS = 0), “SLFN11 low” (HS = 1, 2), “SLFN11 intermediate” (HS = 3, 4), or “SLFN11 high” (HS = 6, 9), see Table 2 [12].

Subsequently, two studies by Winkler et al. were published in 2021, both using the same rabbit polyclonal anti-SLFN11 antibody (ab121731; Abcam, Cambridge, MA, USA) and H-score calculated using an identical methodology (Table 2). In the first study aiming to provide a comprehensive analysis of the clinical significance of SLFN11 as a predictive biomarker to DDA on a wide range of cancer models, including ovarian cancer, the cutoff of 31 H-score showed a good predictive power [46]. On the other hand, in the second study focused on SLFN11 assessment in cohorts of platinum-sensitive (PS; *n* = 15) and platinum-resistant (PR; *n* = 13) ovarian cancer, a H-score cutoff of 60 obtained maximized accuracy in classifying cohorts according to the response to platinum-based chemotherapy. Notably, in this study, SLFN11 transcript levels by quantitative real-time polymerase chain reaction (qRT-PCR) and protein levels in formalin-fixed paraffin-embedded (FFPE) tissue by IHC showed a strongly significant correlation (*p* = 0.0051) [49].

The authors of the study with the largest number of samples were focused on the correlation of SLFN11 tumor levels with the clinical outcomes of patients treated with standard chemotherapy or olaparib maintenance. The whole evaluated cohort of HGSC samples (*n* = 151) consisted of platinum-resistant resection specimens (*n* = 7) obtained from Asterand, samples in a tissue microarray (TMA) format from patients (*n* = 110) treated with olaparib or placebo maintenance in the Study 19 [53], and samples from patients (*n* = 34) with different responses to first-line paclitaxel-carboplatin (PAC/CBDCA) chemotherapy [17]. To divide samples into the ‘SLFN high’ or ‘SLFN-low’ group, an H-score cutoff of 30 was developed based on expression distribution. Interestingly, that value was substantially different from the cohort of patients with small-cell lung cancer in the same study with a H-score cutoff of 122 [52].

Taken together, when assessing the available data about the immunohistochemical analysis of SLFN11 in tumor tissue, including ovarian cancer, the following important facts have to be pointed out. Different types of antibodies, distinct methodologies for evaluating immunohistochemical staining, and most importantly, different cutoffs for SLFN11 positivity have been used in the studies published so far (see Table 2).

The promising method for allowing SLFN11 expression to be tracked longitudinally in a non-invasive manner during the course of the disease seems to be an assessment on circulating tumor cells, as has been described recently in small cell lung cancer [54,55]. However, the potential use of circulating tumor cells in ovarian cancer for this purpose is still unclear. Since the intraperitoneal spread is considered to be the primary way of metastasis in ovarian cancer, circulating tumor cell research has not been too extensive so far in this field. Moreover, their isolation and detection methods affect the use of circulating tumor cells in ovarian cancer [56,57].

## 5. Prevalence of SLFN11 in Ovarian Cancer

According to the findings from preclinical studies, approximately 50% of cancer cell lines do not express SLFN11, making its expression bimodal [35,36]. Besides, the quantification of SLFN11 either by transcript levels or immunohistochemical assessment revealed a broad range of SLFN11 expression in various tumor types, including ovarian cancer [26,29,48,50,52]. These findings indicate potential for the successful use of SLFN11 as a biomarker.

Already in the first study describing the discovery of significant correlation of SLFN11 with the response of cancer cells to DNA-damaging agents, microarray data obtained from patients affected by ovarian carcinoma (*n* = 38) and from corresponding healthy tissues (*n* = 8) available from The Cancer Genome Atlas database were also analyzed. Tumor samples showed a greater than fivefold SLFN11 expression range compared to healthy tissues, with several cancer samples having a higher or lower SLFN11 expression than the interquartile range for its expression in normal tissues (*p*-value < 0.05) [24].

In agreement with preclinical findings, in studies retrospectively evaluating SLFN11 status in samples of patients with ovarian cancer (mainly serous carcinoma) either by IHC or methylation-specific PCR, the proportion of samples with an SLFN11-negative (i.e., IHC 0+; H-score value 0–10) or methylated status counted consistently around 40% [12,25,49,52].

In the comprehensive study by Takashima et al. (see also above in part 4), the evaluated cohort of ovarian cancer and adjacent non-tumor tissue consisted of serous carcinoma (*n* = 18), clear cell carcinoma (*n* = 7), endometrioid carcinoma (*n* = 3), mucinous carcinoma (*n* = 9), and non-tumor Fallopian tube (*n* = 15). Unlike the 100% SLFN11 positivity in non-tumor tissue, a total of 14 cancer samples (38%) were evaluated as SLFN11-negative. A substantial percentage of SLFN11-negative tumors was observed in mucinous carcinoma (77%), while the other histological types showed no SLFN11 expression in 14–33% [29].

Consistent with the findings of Takashima et al., in the retrospective study by Willis et al. evaluating three different cohorts of ovarian cancer samples (see above part 4), the SLFN11 expression was generally low in serous carcinoma with a median H-score of 20. This study described a subset of tumors with sub-clonal SLFN11 expression based on immunohistochemical analysis, where spatially distinct high and low SLFN11 expressing sub-clones were identified within the same patient sample. Sub-clonality was found in 6/34 FFPE blocks of high-grade serous carcinoma from patients with extremely good or poor responses to first-line platinum (P)-based chemotherapy. Still, it was rarely observed in tissue microarrays (TMAs) from the Study 19 cohort due to the small core size. Other SLFN11 protein and RNA detection methods were not suitable for the assessment of sub-clonality [52].

## 6. SLFN11 as a Predictive Biomarker for DDA or PARPi Response in Ovarian Cancer Patients

As mentioned above, the presence of SLFN11 was found to be associated with sensitivity to DDA and PARPi across a variety cancer cell lines and patient-derived xenografts, including small cell lung cancer, colorectal cancer, sarcoma, gastric cancer, mesothelioma, and ovarian cancer. On the other hand, the low or absent expression of SLFN11 has been associated with resistance [30,35]. Remarkably, sensitivity to PARPi seemed to be independent of BRCA1/2 mutations or HR deficiency in preclinical studies [11,45]. Furthermore, the predictive strength of SLFN11 positivity may depend on the degree of PARP trapping that varies among PARPi, with talazoparib being the strongest and veliparib the weakest [30,45].

Based on promising findings from preclinical studies, the predictive role of SLFN11 is being assessed across different cancer types in a clinical setting. There is increasing evidence that patients with SLFN11-positive solid tumors, such as small cell lung cancer or Ewing sarcoma, respond better to DDA and/or PARPi [28,58,59].

Regarding the predictive role of SLFN11 in ovarian cancer, three studies have been published so far, two of them mainly exploratory in nature (see Table 3). Shee et al. analyzed the correlation between tumor SLFN11 mRNA levels and treatment outcomes in the datasets of 75 ovarian cancer patients treated with primary platinum-based chemotherapy. Patients were stratified based on disease-free survival (DFS) as highly sensitive (defined as DFS > 732 days) or non-highly sensitive (non-HS; DFS ≤ 732 days). While statistically limited by the sample size, they demonstrated a trend towards higher levels of SLFN11 expression in highly sensitive vs. non-HS (*p* = 0.057) [20].

In the study by Winkler et al., the internal cohort of 28 patients with advanced stage HGSC was selected based on platinum resistance (*n* = 13; defined as progressing within 6 months from the last dose of platinum-based CT) or sensitivity (*n* = 15) in order to evaluate the predictive value of SLFN11 assessed by IHC. They also evaluated the SLFN11 H-score in cancer cells and in non-cancer stromal cells and overall (i.e., together in cancer and non-cancer cells). While the SLFN11 H-score assessed in cancer cells showed only a borderline significant association (HR = 0.62, 95% confident interval (CI) = 0.38–1.02, *p* = 0.0620), overall and non-cancer higher SLFN11 H-scores were strongly associated with longer progression-free interval (PFI) by univariable analysis (HR = 0.50, 95% CI = 0.33–0.75, *p* = 0.0009; and HR = 0.54, 95% CI = 0.36–0.81, *p* = 0.0028, respectively). Overall SLFN11 protein levels retained their independent prognostic value (adjusted HR = 0.56, 95% CI = 0.37–0.85, *p* = 0.0073), together with age and stage, in the multivariable Cox’s regression model. As the authors concluded, the results suggest that SLFN11 levels in both cancer and non-cancer cells may play a role in response to platinum-containing regimens in HGSC. The statistically significant association of SLFN11 with PFI (HR = 0.68, 95% CI = 0.49–0.95, *p* = 0.0233) was subsequently validated in the TCGA dataset of patients with advanced stage HGSC (*n* = 221; 157 progressive events) [49].

Finally, analyses of another cohort of HGSC patients with extremely good or poor responses to first-line PAC/CBDCA (*n* = 34) published in 2021 by Willis et al. (see also part 4) failed to reveal a significant link between immunohistochemical SLFN11 levels and sensitivity to PAC/CBDCA (*p* = 0.487). Consequently, the differences between median PFI in SLFN11-high patients (14 months) and SLFN11-low patients (6 months) did not reach statistical significance (*p* = 0.0705). The same trend was observed for overall survival (OS) with a median of 103 months in the SLFN11-high group compared to 42 months in the SLFN11-low group (*p* = 0.2475). Remarkably, sub-clonality expression was not associated with clinical outcomes, but patient numbers were limited. Conversely, in the same study, they highlighted a trend of high SLFN11 expression and better clinical outcomes to olaparib in the cohort of patients with platinum-sensitive relapsed HGSC treated with olaparib or a placebo and evaluable for SLFN11 status (*n* = 110). The progression-free survival (PFS) hazard ratio (HR) was lower in the SLFN11-high group (>30 H-score), 0.28 HR [0.09, 0.74 95% CI], compared to the SLFN11-low group, HR 0.49 [0.26, 0.91 95% CI]. In the multivariate analysis with adjustment for BRCA status, median PFS was longest in the SLFN11-high groups treated with olaparib (12.4 months BRCAm and 10 months BRCAwt), compared to the SLFN11-low groups (8.3 months BRCAm and 5.5 months BRCAwt) [52].

## 7. SLFN11 as a Prognostic Biomarker in Ovarian Cancer

The prognostic value of SLFN11 expression has been confirmed in lung [25,52], colorectal [26,60], esophageal [48], gastric [23], bladder cancer [51], and hepatocellular carcinoma [47]. Aside from the studies listed above in part 6, the correlation between SLFN11 expression and prognosis in ovarian cancer has been addressed in two other studies only (see Table 3).

Zoppoli et al. evaluated the expression of SLFN11 by immunoblotting in randomly chosen microarray data obtained from 110 patients affected by ovarian cystadenocarcinoma and treated with a cisplatin-containing regime available from the TCGA database. In the univariate analysis for overall survival, the patients with SLFN11 greater than the mean expression reached a median OS of 80 months [95% CI = 55–105] vs. 49 months [95% CI 38–60] for patients with low SLFN11 expression (*p* = 0.016). SLFN11 retained the significance for OS prediction in a multivariate model together with optimal surgery at diagnosis [24].

Similarly, in the study by Nogales et al. evaluating a cohort of 41 patients with serous ovarian carcinoma treated with platinum compounds, SLFN11 hypermethylation was significantly associated with shorter OS (log-rank test, *p* = 0.006; HR = 3.45; 95% CI= 1.35–8.80). For the patients with PFS information available (*n* = 40), those with SLFN11 hypermethylation showed significantly shorter PFS (*p* = 0.003; HR = 2.99; 95% CI = 1.40–6.40). Moreover, according to the Cox regression multivariate test, SLFN11 was an independent prognostic factor of OS (*p* = 0.02; HR = 2.91; 95% CI = 1.14–7.41) and PFS (*p* = 0.005; HR = 3.35; 95% CI = 1.75–6.42) [25].

In the context of the proven significance of the immune-infiltrating milieu for modulating ovarian cancer prognosis [61,62], the study by Winkler et al. assessed the correlation between not only SLFN11, but also tumor-infiltrating lymphocytes (TILs; evaluated by CD3 and CD8 staining) with outcome in their HGSC patients’ cohort. They found both TILs and SLFN11 to be associated with a better prognosis. As mentioned above in part 6, they assessed not only SLFN11 protein levels in cancer cells but also in different cells of tumor stroma. They yielded exciting evidence of an independent association between SLFN11 density in both the neoplastic and microenvironmental components with a favorable outcome. Taken together, the authors concluded that there is a link between SLFN11 and immune activation in HGSC [49].

## 8. Conclusions and Perspectives

The discovery of biomarkers predicting responses to systemic anticancer treatment remains an unmet clinical need, not only regarding ovarian cancer. SLFN11 plays a vital role in an irreversible cell cycle arrest upon replication stress. It therefore is causally associated with a response to a wide range of DNA-damaging agents, including platinum compounds or PARPi. The data from both preclinical and clinical studies suggest the significant predictive and prognostic value of SLFN11 expression across various cancer types. As SLFN11 expression tends to be very different among organs and tumors or non-tumor tissue, the focus on which organ of origin would be studied seems to be of substantial clinical significance. Although immunohistochemistry seems to be a feasible and most appropriate method for evaluating SLFN11 status in patient samples, there are still ambiguous data regarding the optimal methodology for immunohistochemical assessment, especially in terms of an optimal cut-off for SLFN11 positivity to predict responses to DNA-damaging agents. The proportion of SLFN11-negative tumors in ovarian cancer seems to vary between 15 and40% based on histotype and used assay. Current evidence from the studies published so far exploring the prognostic and predictive role of SLFN11 in ovarian cancer patients is unclear, mainly due to the statistical limitations caused by the small sample size. Data show the association of SLFN11 expression in ovarian cancer cells and stromal cancer cells with a better prognosis of patients treated with platinum-containing regimens. These findings indicate the potential role of SLFN11 as a dual biomarker capturing both immunological and cancer dispositions for the sensitivity to DNA-damaging agents.

Further investigation is needed to obtain consistent and validated data by analyses of large ovarian cancer patient cohorts from real clinical praxis. This should form the basis for establishing a unified algorithm for SLFN11 assays and clarifying the role of SLFN status in the stratification of ovarian cancer patients treated with DNA-damaging agents. Based on proven dynamic changes of SLFN11 expression during tumorigenesis and systemic therapy, the timing of tissue sampling for the predictive purpose as close as possible to the start of treatment or even serial monitoring of SLFN11 in case of subsequent lines of treatment seems to be crucial. Furthermore, prospective studies will be warranted to determine the actual clinical significance of SLFN11 in ovarian cancer. Another promising approach for future studies would be using SLFN11 as a therapeutic target for enhancing sensitivity to anticancer therapy.

## Figures and Tables

**Table 1:** Anticancer agents inducing replication stress (based on [35]).

DNA-Targeting Agents	Representative Drugs	Target	Mechanism of Action
Alkylating agents	Cisplatine Carboplatine Oxaliplatine	DNA template damage	Inter-strand crosslinks
	Temozolomide	DNA template damage MGMT	O^6^-alkyl-guanine lesions on DNA
TOP I and II inhibitors	Irinotecan Topotecan Etoposide Doxorubicin Mitoxantrone	DNA template damage	Block the re-ligation of the TOP-DNA cleavage complexes
PARP inhibitors	Olaparib Rucaparib Niraparib Talazoparib Veliparib	DNA template damage by defective single-strand breaks repair	Generating toxic PARP-DNA complexes
Nucleoside analogs	Gemcitabine Cytarabine 5-azacytidine	DNA elongation inhibition	Blocking DNA polymerase or reducing the pool of nucleotides

Abbreviations: TOP = topoisomerase; PARP = poly (ADP-ribose) polymerase; DNA = deoxyribonucleic acid; MGMT = DNA-repair protein O^6^ methylguanine-DNA methyltransferase.

**Table 2:** Summary of methodologies used for the immunohistochemical analysis of SLFN11 expression in different studies.

Tumor Origin (*n*)	Tissue	Antibody	Evaluation	Author [Reference]
CRC (261)	FFPE	Abcam; ab121731	H-score; value 0–6 ^1^ >4.5—high	Deng [26]
HGSC (75)	*N*/A	*N*/A	H-score; value 0–9 ^2^ 0—negative 1, 2—low 3, 4—intermediate 6, 9—high	Ballestrero [12]
SCLC (12)	PDX	Sigma-Aldrich; HPA023030	H-score; value 0–300 ^3^	Stewart [11]
SCLC (7)	PDX	Sigma-Aldrich; HPA023030	H-score; value 0–300 ^3^ low v. high	Lok [27]
SCLC (48)	FFPE	Sigma-Aldrich; HPA023030	H-score; value 0–300 ^3^ >1—positive	Pietanza [28]
HCC (182/110)	FFPE	Sigma-Aldrich; HPA023030	H-score 0/+—low ++/+++—high	Zhou [47]
TNBC (40)	PDX	Sigma-Aldrich; HPA023030	H-score; value 0–300 ^3^ 0—negative 1–60—low >60—high	Coussy [21]
ESCC (73)	FFPE	Santa Cruz; #sc-515071	H-score; value 0–300 ^3^ ≥51—high	Kagami [48]
16 human adult organs; malignant and adjacent non-tumor tissue (~ 700)	FFPE	Sigma-Aldrich; #H117570 Santa Cruz; #sc-374339 Santa Cruz; #sc-515071	IHC scoring system 1+ (1–10%) 2+ (11–50%) 3+ (51–100%)	Takashima [29]
Various cancer types	PDX	Abcam; ab121731	H-score; value 0–300 ^3^ ≥31—high	Winkler [46]
HGSC (28)	FFPE	Abcam; ab121731	H-score; value 0–300 ^3^ ≥60—high	Winkler [49]
Pediatric sarcoma (220)	FFPE	Sigma-Aldrich; HPA023030	H-score; value 0–300 ^3^ 0—negative ≥1—positive	Gartrell [50]
Gastric (169)	FFPE	Santa Cruz; #sc-515071	IHC scoring system >30%—positive	Takashima [23]
Bladder (120)	FFPE	Santa Cruz; #sc-515071	IHC scoring system >5%—positive	Taniyama [51]
Non-tumor tissue (86) SCLC (124) HGSC (151)	TMA FFPE	Merck; MABF248 Abcam; ab121731 Novus; NBP2–57084	H-score; value 0–300 ^3^ >122—high >30—high	Willis [52]

Abbreviations: *n* = number of patients/samples, CRC = colorectal cancer, OC = ovarian cancer, HGSC = high-grade serous carcinoma, SCLC = small cell lung cancer, TNBC = triple-negative breast cancer, ESCC = esophageal squamous cell carcinoma, DX = patient-derived xenograft, FFPE = Formalin-fixed paraffin-embedded blocks, TMA = tissue microarray. ^1^ HS = intensity (0 … none; 1 … weak; 2 … moderate; 3 … strong) + proportion (0 … <25% positive cells; 1 … 25–50%; 2 … 50–75%; 3 … >75%). ^2^ HS = intensity score (0 … no stain; 1+ … weak; 2+ … moderate; 3+ … intense) × distribution scores (0 ... no stained cells; 1+ … <10%; 2+ … 10–40%; 3+ … >40%). ^3^ HS = (% of cells 1+) + (% of cells 2+) × 2 + (% of cells 1+) × 3.

**Table 3:** Summary of studies evaluating the prognostic or predictive role of SLFN11 in ovarian cancer patients treated with platinum (P)-based chemotherapy or olaparib.

Type	Regime	*n*	Method	Results	Author [Reference]
cystadeno-carcinoma	P-based	110	IB	high SLFN11 expression independently predicts better OS	Zoppoli [24]
serous OC	P-based	41	M	SLFN11 hypermethylation was significantly associated with shorter OS and PFS	Nogales [25]
OC	P-based	110 (OS) 75 (DFS)	RNA-seq	high SLFN11 expression was associated with longer OS trend towards higher levels of SLFN11 expression in patients with DFS > 2y	Shee [20]
HGSC	P-based	28 (PFI) 221 (OS)	IHC	overall H-score in cancer and non-cancer cells was strongly associated with longer PFI SLFN11 is independently prognostic in the TCGA HGSC dataset	Winkler [49]
HGSC	P/T O maint	34 110	IHC	no significant link between SLFN11 levels and sensitivity to P/T trend towards longer PFS and OS in SLFN11-high patients high levels of SLFN11 associated with improved clinical outcome to olaparib	Willis [52]

Abbreviations: OC = ovarian cancer, HGSC = high-grade serous carcinoma, P = platinum, P/T = platinum-paclitaxel, O maint = olaparib maintenance, *n* = number of patients, OS = overall survival, DFS = disease-free survival, PFI = progression-free interval, IB = immunoblotting, M = methylation-specific PCR, IHC = immunohistochemistry, y = years, TCGA = The Cancer Genome Atlas.

## References

[1] Sung H., Ferlay J., Siegel R.L., Laversanne M., Soerjomataram I., Jemal A., Bray F. (2021). Global Cancer Statistics 2020: GLOBOCAN Estimates of Incidence and Mortality Worldwide for 36 Cancers in 185 Countries. CA. Cancer J. Clin..

[2] Cannistra S.A. (2004). Cancer of the Ovary. N. Engl. J. Med..

[3] Colombo N., Sessa C., du Bois A., Ledermann J., McCluggage W.G., McNeish I., Morice P., Pignata S., Ray-Coquard I., Vergote I. (2019). ESMO–ESGO Consensus Conference Recommendations on Ovarian Cancer: Pathology and Molecular Biology, Early and Advanced Stages, Borderline Tumours and Recurrent Disease†. Ann. Oncol..

[4] Wilson M.K., Pujade-Lauraine E., Aoki D., Mirza M.R., Lorusso D., Oza A.M., du Bois A., Vergote I., Reuss A., Bacon M. (2017). Fifth Ovarian Cancer Consensus Conference of the Gynecologic Cancer InterGroup: Recurrent Disease. Ann. Oncol..

[5] Pujade-Lauraine E., Hilpert F., Weber B., Reuss A., Poveda A., Kristensen G., Sorio R., Vergote I., Witteveen P., Bamias A. (2014). Bevacizumab Combined With Chemotherapy for Platinum-Resistant Recurrent Ovarian Cancer: The AURELIA Open-Label Randomized Phase III Trial. J. Clin. Oncol..

[6] Burger R.A., Brady M.F., Bookman M.A., Fleming G.F., Monk B.J., Huang H., Mannel R.S., Homesley H.D., Fowler J., Greer B.E. (2011). Incorporation of Bevacizumab in the Primary Treatment of Ovarian Cancer. N. Engl. J. Med..

[7] Oza A.M., Cook A.D., Pfisterer J., Embleton A., Ledermann J.A., Pujade-Lauraine E., Kristensen G., Carey M.S., Beale P., Cervantes A. (2015). Standard Chemotherapy with or without Bevacizumab for Women with Newly Diagnosed Ovarian Cancer (ICON7): Overall Survival Results of a Phase 3 Randomised Trial. Lancet Oncol..

[8] Aghajanian C., Blank S.V., Goff B.A., Judson P.L., Teneriello M.G., Husain A., Sovak M.A., Yi J., Nycum L.R. (2012). OCEANS: A Randomized, Double-Blind, Placebo-Controlled Phase III Trial of Chemotherapy With or Without Bevacizumab in Patients With Platinum-Sensitive Recurrent Epithelial Ovarian, Primary Peritoneal, or Fallopian Tube Cancer. J. Clin. Oncol..

[9] Colombo I., Kurnit K.C., Westin S.N., Oza A.M. (2018). Moving from Mutation to Actionability. Am. Soc. Clin. Oncol. Educ. Book.

[10] Barretina J., Caponigro G., Stransky N., Venkatesan K., Margolin A.A., Kim S., Wilson C.J., Lehár J., Kryukov G.V., Sonkin D. (2012). The Cancer Cell Line Encyclopedia Enables Predictive Modelling of Anticancer Drug Sensitivity. Nature.

[11] Stewart C.A., Tong P., Cardnell R.J., Sen T., Li L., Gay C.M., Masrorpour F., Fan Y., Bara R.O., Feng Y. (2017). Dynamic Variations in Epithelial-to-Mesenchymal Transition (EMT), ATM, and SLFN11 Govern Response to PARP Inhibitors and Cisplatin in Small Cell Lung Cancer. Oncotarget.

[12] Ballestrero A., Bedognetti D., Ferraioli D., Franceschelli P., Labidi-Galy S.I., Leo E., Murai J., Pommier Y., Tsantoulis P., Vellone V.G. (2017). Report on the First SLFN11 Monothematic Workshop: From Function to Role as a Biomarker in Cancer. J. Transl. Med..

[13] Colombo P.-E., Fabbro M., Theillet C., Bibeau F., Rouanet P., Ray-Coquard I. (2014). Sensitivity and Resistance to Treatment in the Primary Management of Epithelial Ovarian Cancer. Crit. Rev. Oncol. Hematol..

[14] McMullen M., Madariaga A., Lheureux S. (2021). New Approaches for Targeting Platinum-Resistant Ovarian Cancer. Semin. Cancer Biol..

[15] Patch A.-M., Christie E.L., Etemadmoghadam D., Garsed D.W., George J., Fereday S., Nones K., Cowin P., Alsop K., The Australian Ovarian Cancer Study Group (2015). Whole–Genome Characterization of Chemoresistant Ovarian Cancer. Nature.

[16] Lloyd K.L., Cree I.A., Savage R.S. (2015). Prediction of Resistance to Chemotherapy in Ovarian Cancer: A Systematic Review. BMC Cancer.

[17] Weberpals J.I., Pugh T.J., Marco-Casanova P., Goss G.D., Andrews Wright N., Rath P., Torchia J., Fortuna A., Jones G.N., Roudier M.P. (2021). Tumor Genomic, Transcriptomic, and Immune Profiling Characterizes Differential Response to First-line Platinum Chemotherapy in High Grade Serous Ovarian Cancer. Cancer Med..

[18] Tian L., Song S., Liu X., Wang Y., Xu X., Hu Y., Xu J. (2014). Schlafen-11 Sensitizes Colorectal Carcinoma Cells to Irinotecan. Anticancer Drugs.

[19] Kang M.H., Wang J., Makena M.R., Lee J.-S., Paz N., Hall C.P., Song M.M., Calderon R.I., Cruz R.E., Hindle A. (2015). Activity of MM-398, Nanoliposomal Irinotecan (Nal-IRI), in Ewing’s Family Tumor Xenografts Is Associated with High Exposure of Tumor to Drug and High *SLFN11* Expression. Clin. Cancer Res..

[20] Shee K., Wells J.D., Jiang A., Miller T.W. (2019). Integrated Pan-Cancer Gene Expression and Drug Sensitivity Analysis Reveals SLFN11 MRNA as a Solid Tumor Biomarker Predictive of Sensitivity to DNA-Damaging Chemotherapy. PLoS ONE.

[21] Coussy F., El-Botty R., Château-Joubert S., Dahmani A., Montaudon E., Leboucher S., Morisset L., Painsec P., Sourd L., Huguet L. (2020). BRCAness, SLFN11, and RB1 Loss Predict Response to Topoisomerase I Inhibitors in Triple-Negative Breast Cancers. Sci. Transl. Med..

[22] Rathkey D., Khanal M., Murai J., Zhang J., Sengupta M., Jiang Q., Morrow B., Evans C.N., Chari R., Fetsch P. (2020). Sensitivity of Mesothelioma Cells to PARP Inhibitors Is Not Dependent on BAP1 but Is Enhanced by Temozolomide in Cells with High-Schlafen 11 and Low-O6-Methylguanine-DNA Methyltransferase Expression. J. Thorac. Oncol..

[23] Takashima T., Taniyama D., Sakamoto N., Yasumoto M., Asai R., Hattori T., Honma R., Thang P.Q., Ukai S., Maruyama R. (2021). Schlafen 11 Predicts Response to Platinum-Based Chemotherapy in Gastric Cancers. Br. J. Cancer.

[24] Zoppoli G., Regairaz M., Leo E., Reinhold W.C., Varma S., Ballestrero A., Doroshow J.H., Pommier Y. (2012). Putative DNA/RNA Helicase Schlafen-11 (SLFN11) Sensitizes Cancer Cells to DNA-Damaging Agents. Proc. Natl. Acad. Sci. USA.

[25] Nogales V., Reinhold W.C., Varma S., Martinez-Cardus A., Moutinho C., Moran S., Heyn H., Sebio A., Barnadas A., Pommier Y. (2016). Epigenetic Inactivation of the Putative DNA/RNA Helicase SLFN11 in Human Cancer Confers Resistance to Platinum Drugs. Oncotarget.

[26] Deng Y., Cai Y., Huang Y., Yang Z., Bai Y., Liu Y., Deng X., Wang J. (2015). High SLFN11 Expression Predicts Better Survival for Patients with KRAS Exon 2 Wild Type Colorectal Cancer after Treated with Adjuvant Oxaliplatin-Based Treatment. BMC Cancer.

[27] Lok B.H., Gardner E.E., Schneeberger V.E., Ni A., Desmeules P., Rekhtman N., de Stanchina E., Teicher B.A., Riaz N., Powell S.N. (2017). PARP Inhibitor Activity Correlates with SLFN11 Expression and Demonstrates Synergy with Temozolomide in Small Cell Lung Cancer. Clin. Cancer Res..

[28] Pietanza M.C., Waqar S.N., Krug L.M., Dowlati A., Hann C.L., Chiappori A., Owonikoko T.K., Woo K.M., Cardnell R.J., Fujimoto J. (2018). Randomized, Double-Blind, Phase II Study of Temozolomide in Combination with Either Veliparib or Placebo in Patients with Relapsed-Sensitive or Refractory Small-Cell Lung Cancer. J. Clin. Oncol..

[29] Takashima T., Sakamoto N., Murai J., Taniyama D., Honma R., Ukai S., Maruyama R., Kuraoka K., Rajapakse V.N., Pommier Y. (2021). Immunohistochemical Analysis of SLFN11 Expression Uncovers Potential Non-Responders to DNA-Damaging Agents Overlooked by Tissue RNA-Seq. Virchows Arch..

[30] Zhang B., Ramkumar K., Cardnell R.J., Gay C.M., Stewart C.A., Wang W.-L., Fujimoto J., Wistuba I.I., Byers L.A. (2021). A Wake-up Call for Cancer DNA Damage: The Role of Schlafen 11 (SLFN11) across Multiple Cancers. Br. J. Cancer.

[31] Aladjem M.I., Redon C.E. (2017). Order from Clutter: Selective Interactions at Mammalian Replication Origins. Nat. Rev. Genet..

[32] Murai J., Tang S.-W., Leo E., Baechler S.A., Redon C.E., Zhang H., Al Abo M., Rajapakse V.N., Nakamura E., Jenkins L.M.M. (2018). SLFN11 Blocks Stressed Replication Forks Independently of ATR. Mol. Cell.

[33] Mu Y., Lou J., Srivastava M., Zhao B., Feng X.-H., Liu T., Chen J., Huang J. (2016). SLFN11 Inhibits Checkpoint Maintenance and Homologous Recombination Repair. EMBO Rep..

[34] Jo U., Murai Y., Chakka S., Chen L., Cheng K., Murai J., Saha L.K., Miller Jenkins L.M., Pommier Y. (2021). SLFN11 Promotes CDT1 Degradation by CUL4 in Response to Replicative DNA Damage, While Its Absence Leads to Synthetic Lethality with ATR/CHK1 Inhibitors. Proc. Natl. Acad. Sci. USA.

[35] Jo U., Murai Y., Takebe N., Thomas A., Pommier Y. (2021). Precision Oncology with Drugs Targeting the Replication Stress, ATR, and Schlafen 11. Cancers.

[36] Murai J., Thomas A., Miettinen M., Pommier Y. (2019). Schlafen 11 (SLFN11), a Restriction Factor for Replicative Stress Induced by DNA-Targeting Anti-Cancer Therapies. Pharmacol. Ther..

[37] Forment J.V., O’Connor M.J. (2018). Targeting the Replication Stress Response in Cancer. Pharmacol. Ther..

[38] Rajapakse V.N., Luna A., Yamade M., Loman L., Varma S., Sunshine M., Iorio F., Sousa F.G., Elloumi F., Aladjem M.I. (2018). CellMinerCDB for Integrative Cross-Database Genomics and Pharmacogenomics Analyses of Cancer Cell Lines. iScience.

[39] Moribe F., Nishikori M., Takashima T., Taniyama D., Onishi N., Arima H., Sasanuma H., Akagawa R., Elloumi F., Takeda S. (2021). Epigenetic Suppression of SLFN11 in Germinal Center B-Cells during B-Cell Development. PLoS ONE.

[40] Reinhold W.C., Thomas A., Pommier Y. (2017). DNA-Targeted Precision Medicine; Have We Been Caught Sleeping?. Trends Cancer.

[41] Reinhold W.C., Varma S., Sunshine M., Rajapakse V., Luna A., Kohn K.W., Stevenson H., Wang Y., Heyn H., Nogales V. (2017). The NCI-60 Methylome and Its Integration into CellMiner. Cancer Res..

[42] Tang S.-W., Thomas A., Murai J., Trepel J.B., Bates S.E., Rajapakse V.N., Pommier Y. (2018). Overcoming Resistance to DNA-Targeted Agents by Epigenetic Activation of Schlafen 11 (*SLFN11*) Expression with Class I Histone Deacetylase Inhibitors. Clin. Cancer Res..

[43] Gardner E.E., Lok B.H., Schneeberger V.E., Desmeules P., Miles L.A., Arnold P.K., Ni A., Khodos I., de Stanchina E., Nguyen T. (2017). Chemosensitive Relapse in Small Cell Lung Cancer Proceeds through an EZH2-SLFN11 Axis. Cancer Cell.

[44] Lheureux S., Oaknin A., Garg S., Bruce J.P., Madariaga A., Dhani N.C., Bowering V., White J., Accardi S., Tan Q. (2020). EVOLVE: A Multicenter Open-Label Single-Arm Clinical and Translational Phase II Trial of Cediranib Plus Olaparib for Ovarian Cancer after PARP Inhibition Progression. Clin. Cancer Res..

[45] Murai J., Feng Y., Yu G.K., Ru Y., Tang S.-W., Shen Y., Pommier Y. (2016). Resistance to PARP Inhibitors by SLFN11 Inactivation Can Be Overcome by ATR Inhibition. Oncotarget.

[46] Winkler C., Armenia J., Jones G.N., Tobalina L., Sale M.J., Petreus T., Baird T., Serra V., Wang A.T., Lau A. (2021). SLFN11 Informs on Standard of Care and Novel Treatments in a Wide Range of Cancer Models. Br. J. Cancer.

[47] Zhou C., Liu C., Liu W., Chen W., Yin Y., Li C.-W., Hsu J.L., Sun J., Zhou Q., Li H. (2020). SLFN11 Inhibits Hepatocellular Carcinoma Tumorigenesis and Metastasis by Targeting RPS4X via MTOR Pathway. Theranostics.

[48] Kagami T., Yamade M., Suzuki T., Uotani T., Tani S., Hamaya Y., Iwaizumi M., Osawa S., Sugimoto K., Miyajima H. (2020). The First Evidence for SLFN11 Expression as an Independent Prognostic Factor for Patients with Esophageal Cancer after Chemoradiotherapy. BMC Cancer.

[49] Winkler C., King M., Berthe J., Ferraioli D., Garuti A., Grillo F., Rodriguez-Canales J., Ferrando L., Chopin N., Ray-Coquard I. (2021). SLFN11 Captures Cancer-Immunity Interactions Associated with Platinum Sensitivity in High-Grade Serous Ovarian Cancer. JCI Insight.

[50] Gartrell J., Mellado-Largarde M., Clay M.R., Bahrami A., Sahr N.A., Sykes A., Blankenship K., Hoffmann L., Xie J., Cho H.P. (2021). SLFN11 Is Widely Expressed in Pediatric Sarcoma and Induces Variable Sensitization to Replicative Stress Caused By DNA-Damaging Agents. Mol. Cancer Ther..

[51] Taniyama D., Sakamoto N., Takashima T., Takeda M., Pham Q.T., Ukai S., Maruyama R., Harada K., Babasaki T., Sekino Y. (2022). Prognostic Impact of Schlafen 11 in Bladder Cancer Patients Treated with Platinum-based Chemotherapy. Cancer Sci..

[52] Willis S.E., Winkler C., Roudier M.P., Baird T., Marco-Casanova P., Jones E.V., Rowe P., Rodriguez-Canales J., Angell H.K., Ng F.S.L. (2021). Retrospective Analysis of Schlafen11 (SLFN11) to Predict the Outcomes to Therapies Affecting the DNA Damage Response. Br. J. Cancer.

[53] Ledermann J., Harter P., Gourley C., Friedlander M., Vergote I., Rustin G., Scott C., Meier W., Shapira-Frommer R., Safra T. (2012). Olaparib Maintenance Therapy in Platinum-Sensitive Relapsed Ovarian Cancer. N. Engl. J. Med..

[54] Byers L.A., Stewart A., Gay C., Heymach J., Fernandez L., Lu D., Rich R., Chu L., Wang Y., Dittamore R. (2019). Abstract 2215: SLFN11 and EZH2 Protein Expression and Localization in Circulating Tumor Cells to Predict Response or Resistance to DNA Damaging Therapies in Small Cell Lung Cancer. Cancer Res..

[55] Zhang B., Stewart C.A., Gay C.M., Wang Q., Cardnell R., Fujimoto J., Fernandez L., Jendrisak A., Gilbertson C., Schonhoft J. (2021). Abstract 384: Detection of DNA Replication Blocker SLFN11 in Tumor Tissue and Circulating Tumor Cells to Predict Platinum Response in Small Cell Lung Cancer. Cancer Res..

[56] Van Berckelaer C., Brouwers A.J., Peeters D.J.E., Tjalma W., Trinh X.B., van Dam P.A. (2016). Current and Future Role of Circulating Tumor Cells in Patients with Epithelial Ovarian Cancer. Eur. J. Surg. Oncol. EJSO.

[57] Holcakova J., Bartosik M., Anton M., Minar L., Hausnerova J., Bednarikova M., Weinberger V., Hrstka R. (2021). New Trends in the Detection of Gynecological Precancerous Lesions and Early-Stage Cancers. Cancers.

[58] Federico S.M., Pappo A.S., Sahr N., Sykes A., Campagne O., Stewart C.F., Clay M.R., Bahrami A., McCarville M.B., Kaste S.C. (2020). A Phase I Trial of Talazoparib and Irinotecan with and without Temozolomide in Children and Young Adults with Recurrent or Refractory Solid Malignancies. Eur. J. Cancer.

[59] Byers L.A., Bentsion D., Gans S., Penkov K., Son C., Sibille A., Owonikoko T.K., Groen H.J.M., Gay C.M., Fujimoto J. (2021). Veliparib in Combination with Carboplatin and Etoposide in Patients with Treatment-Naïve Extensive-Stage Small Cell Lung Cancer: A Phase 2 Randomized Study. Clin. Cancer Res..

[60] He T., Zhang M., Zheng R., Zheng S., Linghu E., Herman J.G., Guo M. (2017). Methylation of *SLFN11* Is a Marker of Poor Prognosis and Cisplatin Resistance in Colorectal Cancer. Epigenomics.

[61] Hwang W.-T., Adams S.F., Tahirovic E., Hagemann I.S., Coukos G. (2012). Prognostic Significance of Tumor-Infiltrating T Cells in Ovarian Cancer: A Meta-Analysis. Gynecol. Oncol..

[62] Li J., Wang J., Chen R., Bai Y., Lu X. (2017). The Prognostic Value of Tumor-Infiltrating T Lymphocytes in Ovarian Cancer. Oncotarget.

